# Supplementary material for: Analysis of PPARγ Signaling Activity in Psoriasis
Source: Int J Mol Sci. 2021 Aug 10;22(16):8603. doi: 10.3390/ijms22168603 (PMC8395241; doi:10.3390/ijms22168603)
Supplement: Supplementary file 1 [file ijms-22-08603-s001.zip › Supplemental materials_Analysis of PPARg signaling activity in psoriasis/Pathway models/Models images and html files/PPARG inhibits phenotypes assotiated with psoriasis/245193.html]

mucositis


# Disease mucositis

|  |  |
| --- | --- |
| URN | urn:agi-meshdis:Mucositis |
| Total Entities | 2 |
| Connectivity | 1913 |
| Name | mucositis |

---

|  |  |
| --- | --- |
| ChildConcepts | oropharyngeal mucositis |
|  | oral mucositis |

---

|  |  |
| --- | --- |
| Pathway | CFTR Related Ion-Channel Dysfunction in Cystic Fibrosis Airway Epithelium (Class III Mutations) |
|  | PPARG inhibits phenotypes assotiated with psoriasis |

---

|  |  |
| --- | --- |
| MedScan ID | 9018486 |

---

|  |  |
| --- | --- |
| Alias | mucosa inflammations |
|  | irritations of the mucosa |
|  | mucosa inflammation |
|  | inflamed intestinal mucosa |
|  | Mucositides |
|  | mucosa irritations |
|  | Catarrhal Inflammations |
|  | mucus Inflammation |
|  | intestinal mucosa inflammation |
|  | inflammation of the mucosa |
|  | Inflammation mucus Membrane |
|  | Mucosal Inflammation |
|  | Mucitis |
|  | Mucositis |
|  | Mucosal Inflammations |
|  | irritation of the mucosa |
|  | Inflammatory Disease of mucus Membrane |
|  | inflamed mucosa |
|  | mucus Membrane Inflammation |
|  | mucosa irritation |
|  | Catarrhal Inflammation |
|  | Inflammatory Diseases of mucus Membrane |
|  | inflammations of the mucosa |
|  | Inflammation mucus Membranes |
|  | Inflammatory Diseases of Mucous Membrane |
|  | Mucous Membrane Inflammation |
|  | Mucous Inflammation |
|  | Inflammatory Disease of Mucous Membrane |
|  | Inflammation Mucous Membranes |
|  | Inflammation Mucous Membrane |

---

|  |  |
| --- | --- |
| MeSH ID | D052016 |
|  | M0482523 |

---

|  |  |
| --- | --- |
| MeSH Heading | Mucositis |

---
